# Fostering the implementation of transitional care innovations for older persons: prioritizing the influencing key factors using a modified Delphi technique

**DOI:** 10.1186/s12877-021-02672-2

**Published:** 2022-02-16

**Authors:** Amal Fakha, Bram de Boer, Theo van Achterberg, Jan Hamers, Hilde Verbeek

**Affiliations:** 1 CAPHRI Care and Public Health Research Institute, Department of Health Services Research, Faculty of Health, Medicine and Life Sciences, Maastricht University, Maastricht, the Netherlands; 2 Living Lab in Ageing and Long-Term Care, Maastricht, the Netherlands; 3 KU Leuven, Department of Public Health and Primary Care, Academic Centre for Nursing and Midwifery, Kapucijnenvoer 35, 3000 Leuven, Belgium

**Keywords:** Implementation, Innovation, Transitional care, Delphi technique, Leadership, Engagement, Older persons, Factors, Strategies

## Abstract

**Background:**

Transitions in care for older persons requiring long-term care are common and often problematic. Therefore, the implementation of transitional care innovations (TCIs) aims to improve necessary or avert avoidable care transitions. Various factors were recognized as influencers to the implementation of TCIs. This study aims to gain consensus on the relative importance level and the feasibility of addressing these factors with implementation strategies from the perspectives of experts. This work is within TRANS-SENIOR, an innovative research network focusing on care transitions.

**Methods:**

A modified Delphi study was conducted with international scientific and practice-based experts, recruited using purposive and snowballing methods, from multiple disciplinary backgrounds, including implementation science, transitional care, long-term care, and healthcare innovations. This study was built on the findings of a previously conducted scoping review, whereby 25 factors (barriers, facilitators) influencing the implementation of TCIs were selected for the first Delphi round. Two sequential rounds of anonymous online surveys using an a priori consensus level of > 70% and a final expert consultation session were performed to determine the implementation factors’: i) direction of influence, ii) importance, and iii) feasibility to address with implementation strategies. The survey design was guided by the Consolidated Framework for Implementation Research (CFIR). Data were collected using Qualtrics software and analyzed with descriptive statistics and thematic analysis.

**Results:**

Twenty-nine experts from 10 countries participated in the study. Eleven factors were ranked as of the highest importance among those that reached consensus. Notably, organizational and process-related factors, including engagement of leadership and key stakeholders, availability of resources, sense of urgency, and relative priority, showed to be imperative for the implementation of TCIs. Nineteen factors reached consensus for feasibility of addressing them with implementation strategies; however, the majority were rated as difficult to address. Experts indicated that it was hard to rate the direction of influence for all factors.

**Conclusions:**

Priority factors influencing the implementation of TCIs were mostly at the organizational and process levels. The feasibility to address these factors remains difficult. Alternative strategies considering the interaction between the organizational context and the outer setting holds a potential for enhancing the implementation of TCIs.

**Supplementary Information:**

The online version contains supplementary material available at 10.1186/s12877-021-02672-2.

## Background

Transitions in care are common among older persons and entail the movement between different settings and healthcare providers [1, 2]. Research shows that older persons have at least one transition towards their end of life, and one in five experience an adverse event in common transition from hospital to home [1, 3]. Transitional care innovations (TCIs) are emerging evidence-based interventions (EBIs) designed to enhance the continuity and coordination of care for older persons when transferring between multiple care settings [4–6]. Numerous TCIs demonstrated promising evidence for their effectiveness, such as in relation to reducing hospital readmissions, shortening hospital stay, preventing unnecessary admission to a nursing facility, averting hospitalization during an emergency department visit, or improving quality of life [2, 6–10].

While the positive outcomes of TCIs are encouraging, the successful translation of these innovations from trials into “real-world settings” is a main challenge [4, 11]. The implementation of TCIs in practice remains slow and ambiguous with a lack of rigorous evidence on how to best achieve translation [11]. The key components of most TCIs cross the care continuum and involve multiple care settings, which render them intricate and multifaceted [6, 12]. Therefore, integrating TCIs into an existing healthcare system with specific regulations, reimbursement, and funding mechanisms is a starting point of an onerous roadmap to their implementation [11, 13]. Moreover, TCIs normally involve two or more care settings or organizations that can be at different levels of readiness for implementing new interventions. Hence, misalignment of the different organizations’ readiness for change, which encompasses factors such as staff commitment, receptive context for innovation, priority setting, change agents, or dedicated resources, is a basic difficulty in implementation of TCIs [4, 13]. Correspondingly, while the older persons remain the core and common element across various TCIs, the heterogeneity of their care needs prevails. For instance, transitions in care for older persons with dementia [14] differ from those who suffer from the consequences of a stroke [15], which in turn, adds to the complexity of implementing TCIs.

Understanding these challenges and the various interacting constituents of TCIs illuminates the realm of implementing such complex healthcare interventions [16]. Consequently, several research efforts identified factors (barriers, facilitators) influencing the implementation of TCIs in order to better inform implementers and enhance the process [6, 13, 17]. Failure to target the right older population, discontinuous information exchange among care providers, and a lack of organizational resources with low priority given to innovation were among the prominent factors reported to hinder the implementation of TCIs [6]. In contrast, predominant facilitators included a demonstrated advantage of the TCIs for the stakeholders, the presence of frontline staff with a care transition role, as well as a continuous evaluation and monitoring process [6]. However, other factors such as leadership engagement and external policies and incentives were highly reported in the literature, but with a mixed (occasionally enabling/occasionally hindering) influence [6].

Although an awareness of these common factors helps to overcome the challenges of implementing TCIs, this compilation results in multiple and diverse factors, which are highly variable across multiple contexts. Moreover, there is a lack of prioritization based on the importance of the influencing factors, and there is a dearth in evidence on the feasibility of addressing the barriers and leveraging the facilitators with implementation strategies.

This study builds from a scoping review by the research team that identified 25 prominent factors influencing the implementation of TCIs [6]. The study aims to achieve expert consensus on the i) direction of influence (hindering, facilitating) of the known factors relevant to the implementation of TCIs that were predetermined from the literature, ii) the relative degree of importance for each factor in the implementation of TCIs, and iii) the feasibility of addressing each factor with implementation strategies for TCIs.

The overall objective is to derive a prioritized list by degree of importance and feasibility of the factors influencing the implementation of TCIs.

## Methods

All methods used to carry out this study are in accordance with relevant published guidelines and regulations for the Delphi technique, and this report of the study followed the guidelines for reporting Delphi studies (see Additional file 1) [18–21].

### Ethics approval and consent to participate

Ethical approval was granted by the Maastricht University Faculty of Health, Medicine, & Life Sciences Ethics Committee (approval no. FHML-REC/2020/088). Informed consent was obtained from all participants prior to the study and by including a consent statement in each survey round as the initial question, whereby participants needed to agree in order to progress.

### Modified Delphi study approach

A three-step modified Delphi study was conducted with a panel of international experts in the fields of implementation of innovations, transitional care, and long-term care (LTC) between July 2020 and March 2021. The method consisted of two sequential rounds of an online survey and a final group discussion session performed through an online video-conferencing platform.

In this study, the Delphi technique was used as a practical and iterative method to obtain broad perspectives of an experienced mix of experts (in this case, from different countries and, therefore, long-term healthcare system backgrounds) and to achieve consensus in an area where there is not enough evidence [19, 22, 23]. Specifically, a modified Delphi method was chosen. This approach differs from the classical one, because first the content for round one was based on pre-determined items derived from data collected from other resources prior to the Delphi study (in this case, a scoping review) and hence utilized close-ended questions [22–25]. Second, the final round was held as a face-to-face group discussion session with the experts [22, 26–29]. A rating approach was used, whereby a panel of experts anonymously took part in surveys in different rounds. The findings and feedback of round one led the development of round two, and the final expert consultation session was based on input from the previous two rounds [22].

### Participants

The expert profile was defined as individuals with extensive research and/or real-life experience in development, implementation, and evaluation of transitional care innovations (programs, models, and interventions) in LTC settings; healthcare innovations; LTC; or implementation science. Purposive and snowball sampling methods were used to recruit experts internationally.

An initial list of potential experts was developed based on professional contacts of the research team, authors of 21 published studies on the implementation of TCIs (from a previously published scoping review; Fakha et al. 2021) [6], and established contacts from the 3rd UK Implementation Science Conference – July 2020. We aimed for a minimum of 20 participants, as generally recommended [30]. Initially, 62 experts were purposively contacted, and an additional three potential experts were contacted as a result of the snowball technique. All experts were invited to participate in the study by sending them individual recruitment emails along with an invitation letter describing the overall study background, aims, and methodology.

### Data collection

#### Survey design and development

Qualtrics software, an online survey tool, was used to develop and conduct the survey. This entailed sending the different questionnaires of each round to the participants. A total of 25 factors identified from the results of a previously published scoping review study conducted by the research team [6] were used to develop the survey content. Three of these factors were split into two subparts for description clarity, and a final list of 28 factors was thus used in round one (see Table 1). The factors were grouped into the five domains of the Consolidated Framework for Implementation Research (CFIR): *intervention characteristics, outer setting, inner setting, characteristics of individuals, and process* [31]. The survey consisted of three sections and explored the following for each of the 28 factors: i) direction of influence as hindering or facilitating to the implementation of TCIs; ii) importance of influence to the implementation of TCIs; and iii) feasibility (easiness/difficulty) of addressing the factor with implementation strategies for TCIs (see Additional file 2: survey round 1). The survey was piloted among the research team and amended accordingly.

**Table 1:** Predetermined factors (*n* = 28) for Delphi round one grouped into CFIR domains

CFIR Domain	Factors
Intervention (TCIs) Characteristics	▪ **Targeted groups**^**a**^ (older persons as recipients of the TCIs) ▪ **Complexity** (perceived difficulty of TCIs’ implementation) ▪ **Relative advantage** (perceived advantage and usefulness of the TCIs by stakeholders) ▪ **Evidence strength and quality** (evidence for TCIs’ effects on older persons’ outcomes)
Outer Setting	▪ **Cosmopolitanism** (degree to which an organization is networked with other external organizations) ▪ **External policy** (e.g., mandates and regulations supporting the implementation of TCIs) ▪ **External incentives** (e.g., national funding schemes or sponsorship supporting the implementation of TCIs)
Inner Setting^b^	▪ **Networks and communications** (communications within an organization, e.g., interdisciplinary teams) ▪ **Culture** (organizational norms, values) ▪ **Relative priority** (healthcare professionals’/staff’s perception of the importance of the implementation of TCIs) ▪ **Leadership engagement** (commitment and involvement of leaders with the implementation of TCIs) ▪ **Available resources** (resources dedicated to the implementation of TCIs) ▪ **Access to knowledge and information** on the TCIs ▪ **Information continuity**^**a**^ (exchange of medical data on the older person among caregivers and across organizations) ▪ **Health information technology (HIT) systems**^**a**^ (e.g., electronic medical records to manage care)
Characteristics of Individuals	▪ **Knowledge and beliefs of healthcare professionals** about the TCIs ▪ **Knowledge and beliefs of older persons** about the TCIs ▪ **Role**^**a**^ of healthcare professionals/staff in implementing the TCIs ▪ **Skills and competencies**^**a**^ of healthcare professionals/staff involved in implementation of TCIs ▪ **Other personal attributes of healthcare professionals** (values, motivation) ▪ **Other personal attributes of older persons** (values, health literacy)
Process	▪ **Planning** for the implementation of TCIs in advance ▪ **Transition roles of frontline staff**^**a**^ (e.g., transition nurses who will implement the TCIs) ▪ **Reflecting and evaluating** the feedback and progress in the implementation of TCIs ▪ **Measurement capability/data availability**^**a**^ (capacity for the implementation process monitoring, evaluation, and improvement) ▪ **Engaging key stakeholders** (individuals within the organization directly impacted by the TCIs) ▪ **Engaging organizations, external context**^**a**^ (collaborations among various organizations and care providers involved in the implementation of TCIs) ▪ **Engaging innovation participants** (older persons, family, and informal caregivers who participate in the implementation of TCIs)

^a^These factors are constructs from the Care Transitions Framework (CTF), which were added within the CFIR relative domains for the purpose of this study, check scoping review by Fakha et al. 2021 [6] for further details; ^b^Inner setting is also referred to as the organizational context

##### Round one

Personal links to the survey were sent in individual emails to the participants. A consent statement was the initial question, and participants needed to agree in order to progress. Participants were asked to rate each factor on a five-point Likert scale, either in ascending order or from negative to positive [18]. Ratings used per section were as follows: For direction of influence: *1) strongly hindering, 2) hindering, 3) neither hindering nor facilitating, 4) facilitating, 5) strongly facilitating*. For importance of influence: *1) not important, 2) slightly important, 3) moderately important, 4) very important, 5) extremely important*. For feasibility: *1) very difficult, 2) difficult, 3) neither difficult nor easy, 4) easy, 5) very easy*. Moreover, participants were requested to provide additional comments in free-text boxes provided per each section, as well as overall suggestions for additional factors relevant to the implementation of TCIs. The survey required approximately 20 min to complete. Instructions were sent to participants on how to complete the survey, and they were given﻿ two﻿ weeks﻿ to respond. A reminder was sent to participants who did not complete the survey within the two-week period.

##### Round two

Survey round two was conducted online and in a manner similar to that for round one. Only participants who completed round one of the survey were invited to round two. This survey included factors that did not reach consensus from round one and additional factors that were suggested by participants, an established approach using the Delphi technique [22, 23]. The definitions of a few factors were revised based on comments of participants in round one (see Additional file 3: survey round 2). Participants were asked to again rate the factors using the same method as round one, but with knowledge of their individual ratings and the group ratings for each factor from the first round. In addition, a summary report on the results of round one was provided to all participants prior to the second round.

#### Final round: expert consultation session

The final round was comprised of two online video call meetings lasting two hours each, and performed in the same manner and using the same content, through a data-protected, web-based conferencing platform. All participants of round two were invited to join and were assigned to either of the two meetings according to their availability. In order to limit bias, AF and BdB facilitated both sessions, and TvA was an observant who also intervened when necessary to ensure participation from all experts. The sessions’ discussions were recorded and later transcribed. The goal of these sessions was to allow interaction between the experts and provide further clarifications on the overall results from previous rounds. The specific aim, determined by the earlier results, was to i) narrow down the important factors to the “must have” factors, ii) obtain further insights on the feasibility of addressing the important factors, and iii) receive suggestions for potentially relevant implementation strategies. Initially, the results of rounds one and two were presented, and then participants were asked for their overall reflections. Afterwards, two open and predetermined questions were used to guide the discussion in order to allow for further deliberations, as follows:

- What are your views on the top factors? And if you were to choose five “must-have” factors, what would they be?
- Please can you explain why the majority of factors that reached consensus on feasibility (including top important ones) were rated as difficult to address when developing implementation strategies? Any advice on how to approach this? Which strategies would you suggest to tackle each factor?

### Data analysis

Responses were analyzed after the completion of each survey round, and rating scores were calculated as percentages using SPSS Statistics 25 software. Consensus was determined as reached if over 70% of the respondents rated the *i) direction of influence of each factor as either ‘strongly hindering’/‘hindering’ (combined score), ‘strongly facilitating’/‘facilitating’ (combined score), or neither*. Similarly, consensus on the *ii) importance of factors was reached if either combined scores for ‘extremely important’/‘very important’ or combined scores for ‘moderately important’/‘slightly important’ or ‘not important’ were over 70%*, and for *iii) feasibility ‘very easy’/‘easy’, ‘very difficult’/‘difficult’, or ‘neither’*. This level of agreement was used and considered appropriate in previous Delphi studies [23, 24, 32–34]. Free-text comments from rounds one and two were analyzed thematically and discussed among the research team to identify any additional factors or to rephrase and clarify the definitions.

#### Thematic analysis: final expert consultation session

The transcripts of the two final meetings were compiled together and analyzed thematically by three researchers (AF, BdB, TvA) following the six-step method described by Braun and Clarke (2006) [35, 36]. An inductive form of thematic analysis was performed, and the codes created were data-driven. Following data coding, themes were developed and then reviewed iteratively. A thematic map was developed, and a clear delineation of the final themes was discussed and agreed upon by the research team.

## Results

**Fig. 1:**
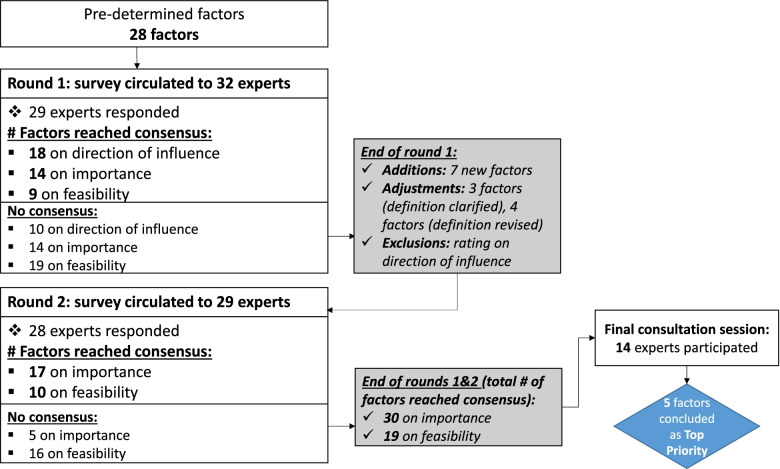
Flow of rounds, participants, and factors through the modified Delphi study^*^. ^*^Final number of factors that reached consensus is a cumulative build up between the consecutive rounds

The overall study design, number of participants, and results per round are summarized in Fig. 1.

### Round one

Initially, 32 experts agreed to participate in the study (round one), out of which 29 responded to the first survey (45% response rate, based on initial number of invited participants). Table 2 presents the participant demographics and professional backgrounds. More than half of the participants had at least 10 years of experience. The majority had a current role in research, mainly in implementation science and transitional care.

**Table 2:** Participant demographics (*n* = 29)

	Frequency
**Country**	
Australia	1
Belgium	1
Canada	2
Germany	1
Netherlands	11
Singapore	1
Sweden	1
Switzerland	1
UK	5
USA	5
**Education level**	
Master’s	4
PhD	25
**Current role**	
Academia/research	26
Practice	5
Both	2
**Area of expertise**^**a**^	
Transitional care	12
Long-term care	11
Healthcare innovations	10
Implementation science	14
**Years of experience**	
3 to 5 years	2
5 to 10 years	10
10 years and above	17

^a^Some participants are experts in more than one area

Eighteen factors out of 28 reached consensus on the direction of influence; however, only one factor (complexity) was generally seen as a barrier, while 17 were seen as facilitators (see Additional file 4). Fourteen factors reached consensus on importance (see Table 3), with all but one factor, rated as very/extremely important in influencing the implementation of TCIs. Engaging key stakeholders ranked as the most important influencing factor. Nine factors reached consensus on feasibility, with only one factor (planning) rated as easy/very easy to address with implementation strategies (see Table 4). The organization’s culture surpassed the other factors as most difficult.

**Table 3:** Factors that reached consensus on importance of influence on the implementation of TCIs, in order of ranking

**Factor**	**Rating: Very/Extremely Important** (Consensus level in %)	**CFIR Domain**
***Round one***		
Engaging key stakeholders	97	Process
Leadership engagement	93	Inner setting
Available resources	93	Inner setting
Relative priority	86	Inner setting
Relative advantage	79	Intervention characteristics
External incentives	79	Outer setting
Transition roles – frontline staff	76	Process
Skills and competencies	72	Characteristics of individuals
Role	72	Characteristics of individuals
Planning	72	Process
Knowledge and beliefs of healthcare professionals about the TCIs	72	Characteristics of individuals
Culture	72	Inner setting
Complexity	72	Intervention characteristics
	**Rating: Slightly/Moderately Important** (Consensus level in %)	
Other personal attributes of older persons	72	Characteristics of individuals
	**Rating: Very/Extremely Important** (Consensus level in %)	
***Round two***		
Leadership engagement^a^	100	Inner setting
Information continuity	96	Inner setting
Financing of TCIs’ implementation	96	Inner/outer setting
HIT systems	93	Inner setting
Access to knowledge and information	89	Inner setting
Engaging organizations, external context	89	Process
Sense of urgency	89	Inner setting
Reflecting and evaluating	86	Process
Other personal attributes of healthcare professionals	82	Characteristics of individuals
Adoption of change in work processes	82	Inner setting
Networks and communications	79	Inner setting
Inter-organizational collaborations	79	Inner/outer setting
Codesign of TCIs	79	Intervention characteristics
Power of decision-makers	75	Inner/outer setting
Measurement capability/data availability	75	Process
External policy	71	Outer setting
	**Rating: Slightly/Moderately Important** (Consensus level in %)	
Evidence strength and quality	71	Intervention characteristics

^a^Definition revised for round two, and therefore rating for this factor was repeated

**Table 4:** Factors that reached consensus on feasibility (easy/difficult to address with implementation strategies), in order of ranking

**Factor**	**Rating: Difficult/Very Difficult** (Consensus level in %)	**CFIR Domain**
***Round one***		
Culture	100	Inner setting
HIT systems	93	Inner setting
Complexity	86	Intervention characteristics
External incentives	83	Outer setting
Networks and communications	76	Inner setting
External policy	76	Outer setting
Available resources	76	Inner setting
Other personal attributes of healthcare professionals	72	Characteristics of individuals
	**Rating: Easy/Very Easy** (Consensus level in %)	
Planning	76	Process
	**Rating: Difficult/Very Difficult** (Consensus level in %)	
***Round two***		
Leadership engagement	93	Inner setting
Engaging organizations, external context	93	Process
Relative priority	86	Inner setting
Information continuity	86	Inner setting
Other personal attributes of older persons	89	Characteristics of individuals
Financing of TCIs’ implementation	89	Inner/outer setting
Cosmopolitanism	82	Outer setting
Adoption of change in work processes	82	Inner setting
	**Rating: Easy/Very Easy** (Consensus level in %)	
Access to knowledge and information	89	Inner setting
	**Neither Easy nor Difficult** (Consensus level in %)	
Transition roles – frontline staff	75	Process

Thematic analysis of the free-text comments in the first round indicated that the direction of influence for the factors was very difficult to assess. The participants mentioned that factors can behave differently according to the specific context where TCIs are implemented. Therefore, it was hard to judge the ultimate influence of each factor. *“Factors that hinder can paradoxically also be factors that facilitate and vice versa” (Expert 8, round 1). “Whether these factors are hindering or facilitating depends highly on the specific nature of this factor in the organization, so culture can be facilitating if there is an innovative culture, but hindering if there is a conservative culture without openness to innovation” (Expert 7, round 1).*

Moreover, the experts identified an additional seven factors to explore for consensus in the consecutive round. These factors were recognized across the five CFIR domains and included power of decision-makers, sense of urgency, adoption of change in work processes, financing of TCIs’ implementation, inter-organizational collaborations, previous experiences with implementation of innovations, and co-design of the TCIs (see Additional file 3: survey round 2). According to the experts’ comments, the definitions of some factors were revised.

### Round two

Twenty-eight of the 29 participants, who completed round one, completed this round (97% response rate). In this round, rating the factors’ direction of influence was omitted. The consensus results from round one were skewed mostly to one direction (facilitating) and hence were ruled as of low relevance and non-conclusive by the research team.

A further nine factors reached consensus as very/extremely important and one as slightly/moderately. Additionally, six out of the seven newly added factors reached consensus as very/extremely important to the implementation of TCIs, with financing of TCIs’ implementation ranked as highest (see Table 3). Ten additional factors achieved consensus on feasibility in this round, with leadership engagement as the most difficult and transition roles as a neutral factor (see Table 4). A further two of the seven new factors (financing of TCIs’ implementation, adoption of change in work processes) reached consensus as difficult/very difficult.

### Factors of highest importance

Of the total 30 factors that reached consensus on the importance of influence following rounds one and two, the majority were linked to the inner setting. Within this domain, leadership engagement, availability of resources including HIT systems, and information continuity between care providers had the highest consensus levels on importance as compared to other factors such as the organizational culture. Whereas the engagement of stakeholders and organizations/external context was of highest importance within the implementation process and exceeded other factors, such as planning, reflecting and evaluating, and transition roles. In comparison, factors (skills and competencies, role, knowledge and beliefs) related to the characteristics of individuals had a lower level of consensus on their importance. Moreover, the personal attributes of older persons such as their motivation, values, or intellectual ability were rated as slightly/moderately important, while factors relating to healthcare professionals were seen as very/extremely important. As for the intervention characteristics, the relative advantage and benefits of the innovation as perceived by stakeholders (older persons and healthcare providers) as well as the degree of involvement of these stakeholders in its design prior to implementation were the most important factors. Alternatively, the demonstrated evidence strength and quality of the TCIs appear to be of least importance to influence the implementation. In contrast, external incentives and policy supporting the TCIs’ implementation and national financing structures, such as a healthcare services reimbursement system, were the important factors within the outer setting.

A final list of the 11 factors that ranked as most important with consensus of 85% and above is presented in Table 5. Once more, these key factors were predominantly related to the inner setting, and only three were linked to the implementation process. The engagement of leaders and key stakeholders was confirmed by the experts as essential in influencing the implementation of TCIs. *“If key stakeholders are in favor of an innovation, good communication can really help, but when they are not in favor, it can really hinder an implementation process” (Expert 7, round 1)*. Nevertheless, the continuity of information and communication across multiple organizations came in third place, which could be explained by the nature of transitional care involving several care settings. *“It is difficult to coordinate care that goes beyond the boundaries of a specific organization” (Expert 7, round 1).* Moreover, the availability of organizational resources as well as the existing financing structures to support the implementation of TCIs were important influencers. *“In transitional care also the reimbursement system in healthcare can play an important role. If an intervention does not fit the current system, this can be a real challenge for the implementation process” (Expert 7, round 1). “Lack of money and lack of management support can stop efforts very quickly” (Expert 5, round 1).*

**Table 5:** Final list of most important factors and indication of feasibility†

Priority Factors*	Feasibility	CFIR Domain
1. **Leadership engagement**	Difficult/very difficult	Inner setting
2. **Engaging key stakeholders**	*No consensus*	Process
3. **Information continuity**	Difficult/very difficult	Inner setting
4. **Financing of TCIs’ implementation**	Difficult/very difficult	Inner/outer setting
5. **Available resources**	Difficult/very difficult	Inner setting
6. **HIT systems**	Difficult/very difficult	Inner setting
7. **Access to knowledge and information**	Easy/very easy	Inner setting
8. **Engaging organizations, external context**	Difficult/very difficult	Process
9. **Sense of urgency**	*No consensus*	Inner setting
10. **Relative priority**	Difficult/very difficult	Inner setting
11. **Reflecting and evaluating**	*No consensus*	Process

†Factors are listed in descending ranking order, *factors with a consensus level ≥ 85% were considered as most important, i.e., priority

### Feasibility

Around only half (54%) of the total number of factors reached consensus on feasibility, with the majority rated as difficult to address with implementation strategies and repeatedly linked to the organizational context. An attempt to address the organizational culture was regarded by experts as topmost difficult and as the least feasible approach to take*. “And since we’re talking about implementing a new model or some sort of a change, it’s always a culture change conversation, and there are a lot of things involved in changing culture...” (Expert 12, consultation session).* Moreover, experts indicated that it was challenging to assess the feasibility for each factor, since it depends on the context where the TCIs’ implementation is happening. *“The difficulty to address these items in practice can vary a lot from project to project” (Expert 5, round 1). “Ideally, each site should identify the areas that are strengths and challenges in relation to the intervention, and from there identify which strengths they can leverage” (Expert 18, round 1).* In addition, consensus on the feasibility of specific factors revealed that, while these factors are very important in influencing the implementation of TCIs, it is most likely difficult to consider, control, or change them with strategies (see Table 5).

### Final round: expert consultation session

Fourteen experts participated in this session and two overarching themes emerged, which are described as follows:

#### Theme one: 'The Catalysts’

This theme describes a combination of temporal and interconnected factors that were seen as essential prerequisites for starting the implementation of TCIs. From the 11 key important factors from the previous rounds, the experts identified five factors that are the catalysts to launch any effort to implement TCIs. These factors were the sense of urgency, relative priority, financing and resources, leadership engagement, and engagement of key stakeholders across the continuum of transitional care. Sense of urgency was identified as a primary factor to induce any change within organizations and even to create priorities and allocate resources needed for the implementation of TCIs. Whereas relative priority was regarded as a *“stop/go”* flag for the implementation of TCIs, it also depends on from whom or where this priority is coming.

*“First to get that sense of urgency and then it realigns priorities.” (Expert 12, consultation session)*

*“And since we're talking about implementing a new model or some sort of a change. It is always a culture change conversation, and there are a lot of things involved in changing culture. Having people feel like this is important; maybe you realize the priorities in their head, but I think the urgency comes first, in my mind.” (Expert 12, consultation session)*

*“So this is the sort of stop/go regardless of leadership engagement, regardless of HIT systems, regardless of result. You are not going to have resources, you are not going to have engagement unless something is the priority, so for me this is almost like a step before.” (Expert 4, consultation session)*

The experts reconfirmed that the engagement of leaders, key stakeholders such as representatives of multiple organizations, older persons, and family caregivers is the backbone for implementing TCIs. However, it is crucial to first identify the role and responsibility of each stakeholder in the implementation process and then to create the right engaging activity and sustain it.

*“Engaging key stakeholders is a means to an end is kind of an initial, you know piece of it is a catalyst for all of the other things that happen up and down those levels.” (Expert 9, consultation session)*

*“…that you have a successful implementation, and that is stakeholder engagement and the leadership engagement.” (Expert 6, consultation session)*

**Fig. 2:**
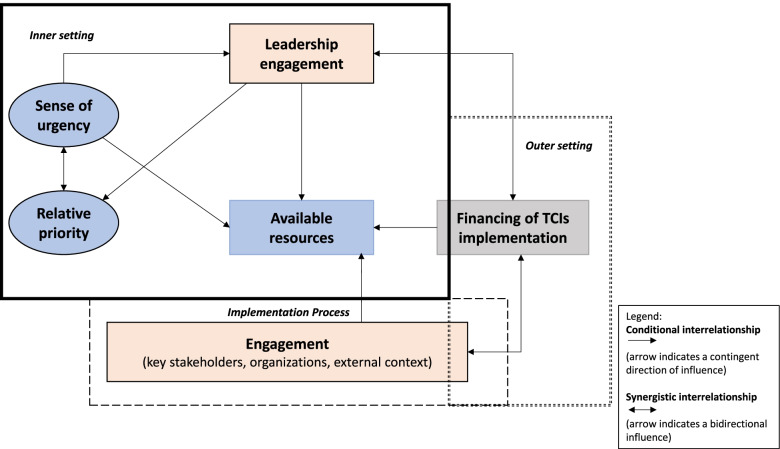
Depiction of the interrelationships among the catalyst factors influencing the implementation of TCIs

Furthermore, it was indicated that resources including HIT systems and funds would only be made available if leadership and key stakeholders are involved early on. As for the factor of financing the implementation of TCIs, it was discussed that reimbursement or financing structures could be varied in transitional care, especially when several organizations are involved. Therefore, the key element is to demonstrate the TCIs’ value for care and the return on investment in order to feedback into the loop of leadership engagement and prioritizing its implementation. The proposed interrelationships among these catalyst factors are depicted in Fig. 2.

#### Theme two: Alignment – 'The Driver'

This theme highlights the importance of aligning the motivation for change across various organizations and levels in the healthcare system. Experts implied that regardless of *‘catalysts’* being present, the alignment of forces to drive the implementation of TCIs across the continuum of transitional care is a key issue, yet often the *“blind spot”*. Aligning the different priorities, interests, motivations, innovation readiness, financial incentives, and agendas of the organizations involved can help drive the implementation of TCIs. Moreover, experts agreed on the significance of considering at which level the implementation should occur and to check if the stakeholders are aligned in their need and motivation for implementing TCIs. Lastly, the experts believed that it is a crucial driver to ensure that the TCIs are in alignment with the older person’s care needs.

*Feasibility –* The experts concurred that developing implementation strategies to address the important factors is largely dependent on the context, individuals involved, and the care continuum. Specifically, trying to overcome hindering factors linked to the organizational context was seen as a known challenge and hard to successfully address with implementation strategies. *“Organizational inertia, culture, its also infrastructure, its processes are inert, is because what they’ve done has worked so far. If they’re surviving it’s because they’ve done something that, for whatever reason, has worked” (Expert 9, consultation session).* However, experts highlighted that the focus could be shifted to creating strategies to induce change at the individual level, which may ultimately improve the organization’s willingness to innovate.

## Discussion

Experts in this study prioritized 11 factors as the most important in the implementation of TCIs. Amongst these factors, the majority were organizational factors, primarily the leadership engagement, availability of resources, information continuity, sense of urgency, and relative priority. Moreover, engagement of stakeholders linked to the implementation process was seen as another priority factor. However, the study results demonstrated a prevalent agreement among experts on the difficulty to address these priority factors with implementation strategies. Nevertheless, ensuring the alignment of the organizations’ interests, agendas, incentives, and priorities was established as a crucial *“stepping stone”* in implementing TCIs across the transitional care continuum. The current findings are congruent with earlier research indicating that organizational factors, chiefly leadership, resources, and communication, significantly influence the implementation of EBIs in healthcare settings [37–40].

In this study, experts concurred strongly that a high commitment of organizational leadership is the dominant factor in initiating the implementation of TCIs and supporting the overall change process. Furthermore, leadership has the ability to respond to a sense of urgency to innovate within an organization and keep it as a priority. Therefore, this suggested a versatile nature of leadership influence on the implementation of TCIs by being not only a precursor but also a probable moderator or mediator. This resonates with recent studies that recognized a contingent relationship between leadership influence and other implementation factors [37, 38, 41]. The other two priority factors — resource allocation and engagement of key stakeholders (i.e., healthcare professionals and staff required for implementation of TCIs) — were acceded by our Delphi panel as dependent on the existence of a supportive leadership influence. Correspondingly, Gifford et al. describe the potential effective contribution of leadership to promote a successful implementation of evidence in healthcare practice [42].

Our panel agreed that engagement of key stakeholders was significant in the implementation of TCIs at all levels of the transitional care continuum. Similarly, engaging multidisciplinary healthcare teams and key staff in various care settings has been widely reported as an integral element and a necessary process activity for implementing innovations in transitional care and LTC in general [6, 38, 39, 43–46]. Nevertheless, despite the well-known importance of stakeholders’ engagement in implementation, there is still vagueness and limited evidence on its definition, who qualifies as a stakeholder, and which best practices to employ [47, 48].

In relation to this, and to our surprise, the importance of engaging the older persons and their family or informal caregivers in the implementation of TCIs was not something our experts reached consensus on. We would have expected that factors related to the older persons including their knowledge, perceptions, attitudes toward and value placed on the TCIs’ services, personal care needs, and an overall consideration of *“what matters to them?”* would be prioritized as very important by the experts. Acknowledging the characteristics and interests of the older persons was revealed as instrumental in other studies describing the process of uptake and implementation of interventions in transitional care [38, 49]. Although person-centered transitional care, whereby TCIs are tailored to older persons’ needs and preferences, is generally seen as important [50]; our results indicate that involving the target group in the implementation of TCIs is less evident. Likewise, Olsen et al. highlight that engaging the older persons and listening to their needs and wishes are fundamental factors in delivering transitional care interventions, yet there appears to be other significant and overlooked constraints at the organizational and system levels [51].

The feasibility of addressing the agreed upon priority factors with implementation strategies was concurred by the experts as mostly challenging, particularly for the organizational factors. Contrary to our expectations, these results do not inform the development of strategies for implementation of TCIs. Notably, a number of taxonomies and compilations of strategies were developed to aide in implementing EBIs in healthcare [52–55]. Moreover, some of these strategies were matched to the relative influencing factors in general healthcare settings [54, 56]. In addition, although organizational leadership was rated as difficult to address in this Delphi study, there is evidence on an emerging strategy: the leadership and organizational change for implementation (LOCI) [57]. Among its aspects, LOCI focuses on leveraging the leaders’ readiness for implementing EBIs, training them on how to overcome implementation barriers, and promoting them to be proactive and create a shared vision within the organization [57]. On the other hand, the body of research on implementation strategies is not specific to transitional care, although it is starting to be utilized in implementing certain TCIs [43]. Therefore, our findings add to the evolving literature by indicating that practitioners and researchers in the field of implementing TCIs perceive that strategies should be tailored to the specific settings involved.

### Implications for implementing innovations in transitional care

#### Practice

In light of our findings, we ask healthcare practitioners (leaders, managers, frontline staff, and other professionals) wanting to implement a TCI, to start by conducting a local needs assessment. It is crucial to know the inner settings of LTC organizations, as well as the inter-organizational differences and dynamics, as this is also a key message from previous studies [39, 58]. To better assess the organizational readiness for putting a TCI into practice, we recommend utilizing our list of ‘priority factors’ as a starting point. Exploring these factors locally will provide an early essential awareness and knowledge of what will most likely help or hinder the implementation of a TCI. Based on the existing literature, we hereby provide hands-on suggestions for addressing the priority factors when implementing a TCI [53, 54, 56]. For example, having both intra/inter-organizational discussions among care providers and key stakeholders can help identify existing problems in care transitions of older persons between different settings, and hence create a sense of urgency or prioritize the need for a TCI as a solution [53, 56]. This, in turn can be used to build a case and to present it to the leaders of LTC organizations, as to obtain support for implementation. Furthermore, engaged leaders can help secure required resources through practical ways by looking for funding options for initial implementation, such as responding for governmental calls to fund implementation of innovations in practice or restructuring organizational incentives. In addition, the creation of inter-organizational working groups of key stakeholders can assist in following through the implementation process of a TCI and making necessary decisions and adjustments [53].

Correspondingly and given the big role of organizational factors, we also highlight the potential value of using insights from four prominent organizational theories *(transaction cost economics, institutional theory, contingency theory, and resource dependency theory)* in implementing TCIs [59]. For example, with keeping in mind the disparities across different healthcare systems, a healthcare manager can assess the transaction cost of implementing an intervention and consider the possibility of outsourcing a TCI’s components or services to another institution. Otherwise as denoted by resource dependency theory, healthcare managers and leaders can establish inter-organizational partnerships and alliances to acquire resources needed. Moreover, as per both institutional and contingency theories, healthcare managers and leaders can promote the adoption and implementation of a TCI within their organization by copying successful innovative behaviours of other organizations in the environment, and boost the organization’s agility to respond to external factors.

#### Future research

Further investigation of the prioritized factors in the actual implementation of TCIs in practice can provide a better understanding of how they exist and interact. In addition, the development and testing of a set of tailored, effective, and feasible strategies to target these priority factors influencing the implementation of TCIs is required.

### Strengths and limitations

This study gathered consensus by drawing on an international panel of experts from the fields of innovation, implementation, and transitional care, which allowed to obtain focused perspectives. Moreover, the use of an online survey permitted a high response, and the final session with the experts was instrumental to understanding the consensus results and enriched the study.

Alternatively, there were some limitations. First, selection bias could play a role. A majority of the panel were scientists rather than professionals from practice, which may have led to an under-representation of insights from real-life context. This could also explain the panel members’ difficulties in assessing implementation factors’ direction of influence and their focus on organizational factors, as their direct care experience might have been limited and/or mainly in the past. Furthermore, the majority of experts came from European healthcare systems. Insights from other alternative models of healthcare systems (e.g. the USA) were under-represented in our panel of experts, thus limiting the applicability of the findings. Second, repeating the survey with other panel experts might have led to other results. However, to improve the reliability, we aimed for a large sample of experts from various backgrounds and countries. Also, we performed the two survey rounds in a consistent manner. Third, we provided the group ratings from round one in the consecutive round, which could be viewed as a pressure to obtain consensus. However, one can also argue that panel members are entitled to receiving core results, besides we followed the Delphi methodology carefully, used findings of a previous literature study to inform the survey, and based our work on an established implementation framework. Lastly, the final round being a qualitative group discussion, holds the limitation pertaining to typical group dynamics and power/confidence of expression. However, the session also enriched our study and facilitators for the session made sure that all participants were heard, and encouraged an open discussion.

## Conclusions

Though many factors are relevant in the implementation of TCIs, experts conceded that the priority factors in the implementation of TCIs are leadership engagement, sense of urgency to innovate, relative priority given to a TCI, availability of organizational resources, and engagement of key stakeholders. Results from our study enable the selection of relevant strategies for implementation of TCIs, yet special attention should be given to inter-organizational factors, which are seen as difficult to address, as well as the interrelationships revealed between these factors. This study provides novel guidance for healthcare researchers and practitioners, opting to improve transitional care for older persons, to better navigate the implementation process of innovations, and deter efforts based on intuition rather than evidence.

## Supplementary Information

**Additional file 1.** CREDES checklist for reporting Delphi studies.

**Additional file 2.** Modified Delphi survey - Round 1.

**Additional file 3.** Modified Delphi survey - Round 2.

**Additional file 4.** List of factors that reached consensus on direction of influence on the implementation of TCIs, from round one.

## Data Availability

Data generated and analyzed during this study are avaliable from the corresponding author on reasonable request.

## Abbreviations

TCI(s): Transitional care innovation(s)
EBIs: Evidence-based interventions
LTC: Long-term care
CFIR: Consolidated Framework for Implementation Research
HIT: Health information technology
LOCI: Leadership and organizational change for implementation
