# Supplementary material for: Fostering the implementation of transitional care innovations for older persons: prioritizing the influencing key factors using a modified Delphi technique
Source: BMC Geriatr. 2022 Feb 16;22:131. doi: 10.1186/s12877-021-02672-2 (PMC8848680; doi:10.1186/s12877-021-02672-2)
Supplement: Supplementary file 2 — Additional file 2. Modified Delphi survey - Round 1. [file 12877_2021_2672_MOESM2_ESM.docx]

Additional file 2: Modified Delphi Study - Survey Round 1

- Dear Expert,

  Thank you for agreeing to participate in the current Modified Delphi study survey (round one) in order to help achieve consensus on the top priority barriers and facilitators that influence the implementation of innovations in transitional care.
- We would like to remind you quickly of key points before starting the survey: ​​>“Transitional care is defined as a set of actions designed to ensure the coordination and continuity of healthcare as patients transfer between different locations or different levels of care within the same location”.

>We refer throughout this survey to Transitional Care (TC) Innovation, as any*intervention, model, or program*  which has been developed with a goal to improve or prevent care transitions for the older population/persons (≥ 65 years old) between different long-term care settings.

- The survey is developed based on the *five domains: (Interventions characteristics, Outer setting, Inner (organizational) setting, Characteristics of Individuals, Process)* of the CFIR (Consolidated Framework for Implementation Research) and selected constructs (factors) from this framework and the CTF (Care Transitions Framework).
- The survey consists of three sections and will explore the following concepts for each of the 25 selected factors (3 factors are split into parts a/b), and definitions of factors adapted to transitional care are provided in the survey:

>Section A: to rate the direction of influence for each of the 25 factors as more a barrier or facilitator to the implementation of transitional care innovations

>Section B: to rate the importance of influence of each of the 25 factors on the implementation of transitional care innovations

>Section C - to indicate the feasibility (easiness / difficulty) to address each of the 25 factors in the development of implementation strategies for transitional care innovations

- *General Instructions:* *The survey will take approximately 15-20 minutes to complete.* *Your participation is voluntary, and you can withdraw from the study during the survey round without comment or penalty by closing the survey link.* *By clicking (yes, I consent) below, you will indicate that you have fully read and understood the complete information document provided to you earlier regarding this study with the participant invitation/recruitment email. Your responses to the survey are automatically saved as you go through the questions, and at any moment you can close the survey link and continue it later by using your same personal link. Please complete the demographics section at the end of the survey.* *All data collected and processed will be kept anonymous, confidential, and stored on a password-protected database at Maastricht University.* *If you have any questions or technical issues please contact: Amal Fakha (main researcher, Department of Health Services Research, Faculty of Health Medicine and Life Sciences, Maastricht University, the Netherlands) by sending an email to a.fakha@maastrichtuniversity.nl.*                           
  On the behalf of the research team we thank you greatly for your participation.
- I hereby agree to participate and undertake this survey.
- Yes, I consent.

|  | | | | | |
| --- | --- | --- | --- | --- | --- |
| **Section A - Direction of Influence:** factors can either hinder or facilitate the implementation of an innovation; for example:  i) a match between the Targeted Groups (factor) and the Transitional Care innovation features can be facilitating to the implementation, while a mismatch can have a hindering effect.  ii) Accessibility to Information and Knowledge (factor) about a Transitional Care innovation can have a facilitating effect on its implementation, while inaccessibility can be hindering.  1) Rate for each of the 25 factors listed below if it acts more often as **hindering or facilitating** to the implementation of Transitional Care Innovations: | | | | | |
|  | Strongly hindering | Hindering | Neither hindering nor facilitating | Facilitating | Strongly facilitating |
| **Domain I - Intervention (Transitional Care Innovation) Characteristics**  **Factor 1 - Targeted Groups:** patients/older population who are the intended recipients or beneficiaries of the transitional care innovation. |  |  |  |  |  |
| **Factor 2 - Complexity:** perceived difficulty of implementation, reflected by duration, scope, radicalness, disruptiveness, centrality, and intricacy and number of steps required to implement. |  |  |  |  |  |
| **Factor 3 - Relative Advantage:** stakeholders’ perception of the advantage (benefits and usefulness) of implementing the transitional care innovation versus an alternative solution. |  |  |  |  |  |
| **Factor 4 - Evidence Strength & Quality**: stakeholders’ perceptions of the quality and validity of evidence (proven effectiveness) supporting the belief that the transitional care intervention will have desired outcomes (e.g. low readmission rates). |  |  |  |  |  |
| **Domain II - Outer Setting**  **Factor 5 - Cosmopolitanism:** the degree to which an organization is networked with other external organizations. |  |  |  |  |  |
| **Factor 6a - External Policy:**a broad construct that includes external strategies (by government or other central entity) to spread transitional care innovations; including policy, regulations, laws, external mandates, legislative changes, recommendations, and guidelines. |  |  |  |  |  |
| **Factor 6b - External Incentives:**a broad construct that includes external strategies (by the government or other central entity) to spread transitional care innovations; including national funding schemes or governmental sponsorship. |  |  |  |  |  |
| **Domain III - Inner (Organizational) Setting**  **Factor 7 - Networks & Communications:** the nature and quality of webs of social networks and of formal/informal communications within an organization (e.g. interdisciplinary teams, coordination & communication among team members). |  |  |  |  |  |
| **Factor 8 - Culture:**norms, values, and basic assumptions of a given organization. |  |  |  |  |  |
| **Factor 9 - Relative Priority:**individuals’ (healthcare professionals, staff within implementing team) shared perception of the importance of the implementation of a transitional care innovation within the organization. |  |  |  |  |  |
| **Factor 10 - Leadership Engagement:**commitment, involvement, and accountability of leaders and managers with the implementation of a transitional care innovation. |  |  |  |  |  |
| **Factor 11 - Available Resources:**the level of resources dedicated for the implementation and on-going operations of a transitional care innovation; including staffing levels, money, funding, training, education, physical space, equipment, and time. |  |  |  |  |  |
| **Factor 12 - Access to Knowledge & Information:**ease of access to digestible information and knowledge (e.g. mentoring, initial training) about the transitional care innovation and how to incorporate it into work tasks. |  |  |  |  |  |
| **Factor 13 - Continuity:**information continuity (e.g. patient information exchange, services & care planning) and relationship continuity, both with providers and patients/caregivers and across organizations. |  |  |  |  |  |
| **Factor 14 - IT&HIT resources (HIT systems):**electronic information management infrastructure and technologies (e.g. electronic health records) available to clinicians to manage patient care, data, and communications. |  |  |  |  |  |
| **Domain IV - Characteristics of Individuals**  **Factor 15a - Knowledge & Beliefs about the Intervention: healthcare professionals/staff** within implementing team's beliefs, expectations, and familiarity with facts, truths, & principles related to the transitional care innovation. |  |  |  |  |  |
| **Factor 15b - Knowledge & Beliefs about the Intervention: patients/older persons'** attitudes toward and value placed on the transitional care innovation as well as awareness on its care services & goals. |  |  |  |  |  |
| **Factor 16 - Role:**individual’s role (healthcare professionals, staff within implementing team) and responsibility for the transitional care innovation; including the degree of multiple or shared roles. |  |  |  |  |  |
| **Factor 17 - Skills & Competencies:**degree of relevant subject matter expertise, skills, and competencies within the implementing team, unit, and organization. |  |  |  |  |  |
| **Factor 18a - Other Personal Attributes: healthcare professionals'**other personal traits such as motivation levels, values, tolerance of ambiguity, critical attributes, intellectual ability, capacity, and learning style. |  |  |  |  |  |
| **Factor 18b - Other Personal Attributes: patients/older persons'**other personal traits such as health literacy, values, and acknowledgement of own care needs. |  |  |  |  |  |
| **Domain V - (Implementation) Process**  **Factor 19 - Planning:** the degree to which a scheme or method of behavior and tasks for implementing an innovation are developed in advance, and the quality of those schemes or methods. |  |  |  |  |  |
| **Factor 20 - Transition Roles (Frontline Staff):** administrative staff, providers (within and outside the organization), e.g. frontline staff such as transition nurses or advanced practice nurses with designated transition roles who will carry out the innovation or be affected by it. |  |  |  |  |  |
| **Factor 21 - Reflecting & Evaluating:**quantitative and qualitative feedback about the progress and quality of implementation accompanied with regular personal and team debriefing about progress and experience. |  |  |  |  |  |
| **Factor 22**- **Measurement Capability/Data Availability:**availability of timely data. Capacity for monitoring, evaluation, and process improvement. Includes measurement differences; accountability for collection, documentation, and analysis. |  |  |  |  |  |
| **Factor 23**- **Engaging Key Stakeholders:**individuals from within the organization that are directly impacted by the transitional care innovation, e.g. staff responsible for making referrals to a new program or using a new work process. |  |  |  |  |  |
| **Factor 24**- **Engaging Organizations, External Context:**developing and capitalizing on relationships with healthcare professionals and frontline staff in the implementing organizations, and promoting external collaborations with outside care providers, and resources linked to the implementation of a transitional care innovation. |  |  |  |  |  |
| **Factor 25**- **Engaging Innovation Participants:**individuals (patients/older persons, family, informal caregivers) served by the organization that participate in the transitional care innovation. |  |  |  |  |  |

Q1) Please provide any further comments you might have on the direction of influence of the 25 factors in relation to implementing Transitional Care Innovations:

________________________________________________________________

________________________________________________________________

________________________________________________________________

________________________________________________________________

_______________________________________________________________

|  | | | | | |
| --- | --- | --- | --- | --- | --- |
| **Section B - Importance of Influence:**      2) Rate the **importance of influence** of each of the 25 factors listed below on the implementation of Transitional Care Innovations: | | | | | |
|  | Not important | Slightly important | Moderately important | Very important | Extremely important |
| **Domain I - Intervention (Transitional Care Innovation) Characteristics**  **Factor 1 - Targeted Groups:** patients/older population who are the intended recipients or beneficiaries of the transitional care innovation. (1) |  |  |  |  |  |
| **Factor 2 - Complexity:** perceived difficulty of implementation, reflected by duration, scope, radicalness, disruptiveness, centrality, and intricacy and number of steps required to implement.   (2) |  |  |  |  |  |
| **Factor 3 - Relative Advantage:** stakeholders’ perception of the advantage (benefits and usefulness) of implementing the transitional care innovation versus an alternative solution. (3) |  |  |  |  |  |
| **Factor 4 - Evidence Strength & Quality**: stakeholders’ perceptions of the quality and validity of evidence (proven effectiveness) supporting the belief that the transitional care intervention will have desired outcomes (e.g. low readmission rates). (4) |  |  |  |  |  |
| **Domain II - Outer Setting Factor 5 - Cosmopolitanism:** the degree to which an organization is networked with other external organizations.  (5) |  |  |  |  |  |
| **Factor 6a - External Policy:**a broad construct that includes external strategies (by government or other central entity) to spread transitional care innovations; including policy, regulations, laws, external mandates, legislative changes, recommendations, and guidelines.   (6) |  |  |  |  |  |
| **Factor 6b - External Incentives:**a broad construct that includes external strategies (by the government or other central entity) to spread transitional care innovations; including national funding schemes or governmental sponsorship. (27) |  |  |  |  |  |
| **Domain III - Inner (Organizational) Setting Factor 7 - Networks & Communications:** the nature and quality of webs of social networks and of formal/informal communications within an organization (e.g. interdisciplinary teams, coordination & communication among team members). (7) |  |  |  |  |  |
| **Factor 8 - Culture:**norms, values, and basic assumptions of a given organization. (8) |  |  |  |  |  |
| **Factor 9 - Relative Priority:**individuals’ (healthcare professionals, staff within implementing team) shared perception of the importance of the implementation of a transitional care innovation within the organization. (9) |  |  |  |  |  |
| **Factor 10 - Leadership Engagement:**commitment, involvement, and accountability of leaders and managers with the implementation of a transitional care innovation. (10) |  |  |  |  |  |
| **Factor 11 - Available Resources:**the level of resources dedicated for the implementation and on-going operations of a transitional care innovation; including staffing levels, money, funding, training, education, physical space, equipment, and time. (11) |  |  |  |  |  |
| **Factor 12 - Access to Knowledge & Information:**ease of access to digestible information and knowledge (e.g. mentoring, initial training) about the transitional care innovation and how to incorporate it into work tasks. (12) |  |  |  |  |  |
| **Factor 13 - Continuity:**information continuity (e.g. patient information exchange, services & care planning) and relationship continuity, both with providers and patients/caregivers and across organizations. (13) |  |  |  |  |  |
| **Factor 14 - IT&HIT resources (HIT systems):**electronic information management infrastructure and technologies (e.g. electronic health records) available to clinicians to manage patient care, data, and communications. (14) |  |  |  |  |  |
| **Domain IV - Characteristics of Individuals**  **Factor 15a - Knowledge & Beliefs about the Intervention: healthcare professionals/staff** within implementing team's beliefs, expectations, and familiarity with facts, truths, & principles related to the transitional care innovation.   (15) |  |  |  |  |  |
| **Factor 15b - Knowledge & Beliefs about the Intervention: patients/older persons'** attitudes toward and value placed on the transitional care innovation as well as awareness on its care services & goals. (28) |  |  |  |  |  |
| **Factor 16 - Role:**individual’s role (healthcare professionals, staff within implementing team) and responsibility for the transitional care innovation; including the degree of multiple or shared roles. (16) |  |  |  |  |  |
| **Factor 17 - Skills & Competencies:**degree of relevant subject matter expertise, skills, and competencies within the implementing team, unit, and organization. (17) |  |  |  |  |  |
| **Factor 18a - Other Personal Attributes: healthcare professionals'**other personal traits such as motivation levels, values, tolerance of ambiguity, critical attributes, intellectual ability, capacity, and learning style.  (18) |  |  |  |  |  |
| **Factor 18b - Other Personal Attributes: patients/older persons'**other personal traits such as health literacy, values, and acknowledgement of own care needs. (29) |  |  |  |  |  |
| **Domain V - (Implementation) Process Factor 19 - Planning:** the degree to which a scheme or method of behavior and tasks for implementing an innovation are developed in advance, and the quality of those schemes or methods. (19) |  |  |  |  |  |
| **Factor 20 - Transition Roles (Frontline Staff):** administrative staff, providers (within and outside the organization), e.g. frontline staff such as transition nurses or advanced practice nurses with designated transition roles who will carry out the innovation or be affected by it. (20) |  |  |  |  |  |
| **Factor 21 - Reflecting & Evaluating:**quantitative and qualitative feedback about the progress and quality of implementation accompanied with regular personal and team debriefing about progress and experience. (21) |  |  |  |  |  |
| **Factor 22**- **Measurement Capability/Data Availability:**availability of timely data. Capacity for monitoring, evaluation, and process improvement. Includes measurement differences; accountability for collection, documentation, and analysis. (22) |  |  |  |  |  |
| **Factor 23**- **Engaging Key Stakeholders:**individuals from within the organization that are directly impacted by the transitional care innovation, e.g. staff responsible for making referrals to a new program or using a new work process. (23) |  |  |  |  |  |
| **Factor 24**- **Engaging Organizations, External Context:**developing and capitalizing on relationships with healthcare professionals and frontline staff in the implementing organizations, and promoting external collaborations with outside care providers, and resources linked to the implementation of a transitional care innovation. (24) |  |  |  |  |  |
| **Factor 25**- **Engaging Innovation Participants:**individuals (patients/older persons, family, informal caregivers) served by the organization that participate in the transitional care innovation. (25) |  |  |  |  |  |

Q2) Please provide any further comments you might have on the importance of influence of the 25 factors in relation to implementing Transitional Care Innovations:

________________________________________________________________

________________________________________________________________

________________________________________________________________

________________________________________________________________

________________________________________________________________

|  | | | | | |
| --- | --- | --- | --- | --- | --- |
| **Section C - Feasibility (easiness/difficulty):**     3) How **easy/difficult** is it to address each of the 25 factors listed below in the development of implementation strategies for Transitional Care Innovations? | | | | | |
|  | Very difficult | Difficult | Neither difficult nor easy | Easy | Very easy |
| **Domain I - Intervention (Transitional Care Innovation) Characteristics**  **Factor 1 - Targeted Groups:** patients/older population who are the intended recipients or beneficiaries of the transitional care innovation. |  |  |  |  |  |
| **Factor 2 - Complexity:** perceived difficulty of implementation, reflected by duration, scope, radicalness, disruptiveness, centrality, and intricacy and number of steps required to implement. |  |  |  |  |  |
| **Factor 3 - Relative Advantage:** stakeholders’ perception of the advantage (benefits and usefulness) of implementing the transitional care innovation versus an alternative solution. |  |  |  |  |  |
| **Factor 4 - Evidence Strength & Quality**: stakeholders’ perceptions of the quality and validity of evidence (proven effectiveness) supporting the belief that the transitional care intervention will have desired outcomes (e.g. low readmission rates). |  |  |  |  |  |
| **Domain II - Outer Setting Factor 5 - Cosmopolitanism:** the degree to which an organization is networked with other external organizations. |  |  |  |  |  |
| **Factor 6a - External Policy:**a broad construct that includes external strategies (by government or other central entity) to spread transitional care innovations; including policy, regulations, laws, external mandates, legislative changes, recommendations, and guidelines. |  |  |  |  |  |
| **Factor 6b - External Incentives:**a broad construct that includes external strategies (by the government or other central entity) to spread transitional care innovations; including national funding schemes or governmental sponsorship. |  |  |  |  |  |
| **Domain III - Inner (Organizational) Setting Factor 7 - Networks & Communications:** the nature and quality of webs of social networks and of formal/informal communications within an organization (e.g. interdisciplinary teams, coordination & communication among team members). |  |  |  |  |  |
| **Factor 8 - Culture:**norms, values, and basic assumptions of a given organization. |  |  |  |  |  |
| **Factor 9 - Relative Priority:**individuals’ (healthcare professionals, staff within implementing team) shared perception of the importance of the implementation of a transitional care innovation within the organization. |  |  |  |  |  |
| **Factor 10 - Leadership Engagement:**commitment, involvement, and accountability of leaders and managers with the implementation of a transitional care innovation. |  |  |  |  |  |
| **Factor 11 - Available Resources:**the level of resources dedicated for the implementation and on-going operations of a transitional care innovation; including staffing levels, money, funding, training, education, physical space, equipment, and time. |  |  |  |  |  |
| **Factor 12 - Access to Knowledge & Information:**ease of access to digestible information and knowledge (e.g. mentoring, initial training) about the transitional care innovation and how to incorporate it into work tasks. |  |  |  |  |  |
| **Factor 13 - Continuity:**information continuity (e.g. patient information exchange, services & care planning) and relationship continuity, both with providers and patients/caregivers and across organizations. |  |  |  |  |  |
| **Factor 14 - IT&HIT resources (HIT systems):**electronic information management infrastructure and technologies (e.g. electronic health records) available to clinicians to manage patient care, data, and communications. |  |  |  |  |  |
| **Domain IV - Characteristics of Individuals**  **Factor 15a - Knowledge & Beliefs about the Intervention: healthcare professionals/staff** within implementing team's beliefs, expectations, and familiarity with facts, truths, & principles related to the transitional care innovation. |  |  |  |  |  |
| **Factor 15b - Knowledge & Beliefs about the Intervention: patients/older persons'** attitudes toward and value placed on the transitional care innovation as well as awareness on its care services & goals. |  |  |  |  |  |
| **Factor 16 - Role:**individual’s role (healthcare professionals, staff within implementing team) and responsibility for the transitional care innovation; including the degree of multiple or shared roles. |  |  |  |  |  |
| **Factor 17 - Skills & Competencies:**degree of relevant subject matter expertise, skills, and competencies within the implementing team, unit, and organization. |  |  |  |  |  |
| **Factor 18a - Other Personal Attributes: healthcare professionals'**other personal traits such as motivation levels, values, tolerance of ambiguity, critical attributes, intellectual ability, capacity, and learning style. |  |  |  |  |  |
| **Factor 18b - Other Personal Attributes: patients/older persons'**other personal traits such as health literacy, values, and acknowledgement of own care needs. |  |  |  |  |  |
| **Domain V - (Implementation) Process Factor 19 - Planning:** the degree to which a scheme or method of behavior and tasks for implementing an innovation are developed in advance, and the quality of those schemes or methods. |  |  |  |  |  |
| **Factor 20 - Transition Roles (Frontline Staff):** administrative staff, providers (within and outside the organization), e.g. frontline staff such as transition nurses or advanced practice nurses with designated transition roles who will carry out the innovation or be affected by it. |  |  |  |  |  |
| **Factor 21 - Reflecting & Evaluating:**quantitative and qualitative feedback about the progress and quality of implementation accompanied with regular personal and team debriefing about progress and experience. |  |  |  |  |  |
| **Factor 22**- **Measurement Capability/Data Availability:**availability of timely data. Capacity for monitoring, evaluation, and process improvement. Includes measurement differences; accountability for collection, documentation, and analysis. |  |  |  |  |  |
| **Factor 23**- **Engaging Key Stakeholders:**individuals from within the organization that are directly impacted by the transitional care innovation, e.g. staff responsible for making referrals to a new program or using a new work process. |  |  |  |  |  |
| **Factor 24**- **Engaging Organizations, External Context:**developing and capitalizing on relationships with healthcare professionals and frontline staff in the implementing organizations, and promoting external collaborations with outside care providers, and resources linked to the implementation of a transitional care innovation. |  |  |  |  |  |
| **Factor 25**- **Engaging Innovation Participants:**individuals (patients/older persons, family, informal caregivers) served by the organization that participate in the transitional care innovation. |  |  |  |  |  |

Q3) Please provide any further comments you might have on the feasibility (easiness/difficulty) to address each of the 25 factors in relation to implementing Transitional Care Innovations:

________________________________________________________________

________________________________________________________________

________________________________________________________________

________________________________________________________________

________________________________________________________________

Q4) Please provide any additional factors (barriers or facilitators) that you would consider important/relevant to the implementation of Transitional Care Innovations.

________________________________________________________________

________________________________________________________________

________________________________________________________________

**Demographics:**

1) Country (current place of residence/work):

- Netherlands
- Switzerland
- United Kingdom
- Sweden
- United States of America
- Canada
- Australia
- Singapore
- Other ________________________________________________

2) Education level:

- Masters level
- PhD level

3) Current role:

- Researcher
- Professor
- Associate Professor
- Assistant Professor
- Lecturer
- Practitioner, please specify ___________________
- Manager, please specify _____________________
- Director, please specify ______________________
- Other ____________________________

4) Please indicate in which field you are an expert (choose all that applies):

- Transitional care
- Long-term care
- Healthcare innovations
- Implementation science

5) Please indicate the total years of experience in your field of expertise:

- 3 to 5 years
- 5 to 10 years
- 10 years and above
