# Supplementary material for: Fostering the implementation of transitional care innovations for older persons: prioritizing the influencing key factors using a modified Delphi technique
Source: BMC Geriatr. 2022 Feb 16;22:131. doi: 10.1186/s12877-021-02672-2 (PMC8848680; doi:10.1186/s12877-021-02672-2)
Supplement: Supplementary file 3 — Additional file 3. Modified Delphi survey - Round 2. [file 12877_2021_2672_MOESM3_ESM.docx]

Additional file 3: Modified Delphi Study - Survey Round 2

- Dear Expert,

  Thank you for participating in the current Modified Delphi study survey and completing round 1. Hereby, we start round 2 of the study in order to reach unattained consensus from round 1 on the barriers & facilitators that influence the implementation of innovations in transitional care.
- Please refer to the results report - round 1 that was sent to you by email in order to obtain further information on the results from the first survey.
- We would like to remind you again of key points before starting the survey: ​​>“Transitional care is defined as a set of actions designed to ensure the coordination and continuity of healthcare as patients transfer between different locations or different levels of care within the same location”.

>We refer throughout this survey to Transitional Care (TC) Innovation, as *any intervention, model, or program*, which has been developed with a goal to improve or prevent care transitions for the older population/persons (≥ 65 years old) between different long-term care settings.

- The survey is developed based on the *five domains: (Interventions characteristics, Outer setting, Inner (organizational) setting, Characteristics of Individuals, Process)* of the CFIR (Consolidated Framework for Implementation Research) and selected constructs (factors) from this framework and the CTF (Care Transitions Framework).
- This survey consists of two sections and will explore the following concepts for each of the factors that didn't reach consensus in round 1, in addition to 7 new factors suggested by the respondents from round 1:

>Section I - to rate the importance of influence of each factor on the implementation of transitional care innovations

>Section II - to indicate the feasibility (easiness/difficulty) to address each factor in the development of implementation strategies for transitional care innovations

- *General Instructions:* *The survey will take approximately 15-20 minutes to complete.* *Your participation is voluntary, and you can withdraw from the study during the survey round without comment or penalty by closing the survey link. By clicking (yes, I consent) below, you will indicate that you have fully read and understood the complete information document provided to you earlier regarding this study with the participant invitation/recruitment email. Your responses to the survey are automatically saved as you go through the questions, and at any moment you can close the survey link and continue it later by using your same personal link. All data collected and processed will be kept anonymous, confidential, and stored on a password-protected database at Maastricht University.* *If you have any questions or technical issues please contact: Amal Fakha (main researcher, Department of Health Services Research, Faculty of Health Medicine and Life Sciences, Maastricht University, the Netherlands) by sending an email to*[*a.fakha@maastrichtuniversity.nl*](mailto:a.fakha@maastrichtuniversity.nl)*.*                           
  On the behalf of the research team, we thank you greatly for your participation.
- I hereby agree to participate and undertake this survey.
- Yes, I consent.

**Section I - Importance of Influence:** determining the level of importance of the influence of each factor on the implementation of a transitional care innovation can be complicated due to the overall context and the interaction among various factors in the process; yet it remains crucial. Therefore, utilize your experience in the current healthcare field in order to respond to this section.  
  
The factors listed below are those that did not reach consensus in round 1 (except for leadership engagement which is redefined in this round), in addition to 7 new factors suggested by the respondents from round 1.

>Please, for each factor review the group choices & your individual choice from round 1 and re-rate the **importance of influence** of each on the implementation of Transitional Care Innovations. Also, you may decide to keep your original choice that you had from round.

>Experts' comments for some factors from round 1 are provided where available & applicable, in order to clarify further their definition and interpretation.  For the new factors, please rate accordingly.

>Group choices are provided as % out of 100% = 29 (total number of respondents in round.

>Note: for some factors their definitions were revised for this round (based on expert comments the definition is rephrased and enhanced to maximize clarity), in this case a label (revised) is added only next to those factors whose definitions were enhanced.

**Please, note that the group choices, individual choices percentages, and experts’ comments were omitted from this additional file, for data protection purposes.*

|  | Not important | Slightly important | Moderately important | Very important | Extremely important |
| --- | --- | --- | --- | --- | --- |
| **Domain I - Intervention (Transitional Care Innovation) Characteristics Factor 1 - Targeted Groups (revised):** patients/older population who are the intended recipients or beneficiaries of the transitional care innovation (e.g. matching the care needs of older persons with high frailty or dementia). |  |  |  |  |  |
| **Factor 4 - Evidence Strength & Quality**: stakeholders’ perceptions of the quality and validity of evidence (proven effectiveness) supporting the belief that the transitional care intervention will have desired outcomes (e.g. low readmission rates). |  |  |  |  |  |
| **Domain II - Outer Setting Factor 5 - Cosmopolitanism (revised):** the degree to which an organization is networked with other external organizations (e.g. pre-existing partnerships; sharing of healthcare practices with external organizations). |  |  |  |  |  |
| **Factor 6a - External Policy:**a broad construct that includes external strategies (by government or other central entity) to spread transitional care innovations; including policy, regulations, laws, external mandates, legislative changes, recommendations, and guidelines. |  |  |  |  |  |
| **Domain III - Inner (Organizational) Setting Factor 7 - Networks & Communications:** the nature and quality of webs of social networks and of formal/informal communications within an organization (e.g. interdisciplinary teams, coordination & communication among team members). |  |  |  |  |  |
| **Factor 10 - Leadership Engagement (revised):**commitment, involvement, and accountability of leaders & managers with the implementation of a transitional care innovation. In addition, the presence of a skilled, motivated, and continuous leadership throughout the implementation (e.g. minimal turnover of dedicated project managers with high interest in the implementation). |  |  |  |  |  |
| **Factor 12 - Access to Knowledge & Information:**ease of access to digestible information and knowledge (e.g. mentoring, initial training) about the transitional care innovation and how to incorporate it into work tasks. |  |  |  |  |  |
| **Factor 13 - Continuity (revised):**care transitions' information continuity (e.g. exchange of patient medical information and the services & care plans between healthcare providers). In addition, the continuity of steady work relationships between the healthcare providers and patients/caregivers and across all the organizations involved in the transitional care innovation implementation. |  |  |  |  |  |
| **Factor 14 - IT&HIT resources (HIT systems):**electronic information management infrastructure and technologies (e.g. electronic health records) available to clinicians to manage patient care, data, and communications. |  |  |  |  |  |
| **Domain IV - Characteristics of Individuals**  **Factor 15b - Knowledge & Beliefs about the Intervention: patients/older persons'** attitudes toward and value placed on the transitional care innovation as well as awareness on its care services & goals. |  |  |  |  |  |
| **Factor 18a - Other Personal Attributes: healthcare professionals'**other personal traits such as motivation levels, values, tolerance of ambiguity, critical attributes, intellectual ability, capacity, and learning style. |  |  |  |  |  |
| **Domain V - (Implementation) Process** **Factor 21 - Reflecting & Evaluating:**quantitative and qualitative feedback about the progress and quality of implementation accompanied with regular personal and team debriefing about progress and experience. |  |  |  |  |  |
| **Factor 22**- **Measurement Capability/Data Availability:**availability of timely data. Capacity for monitoring, evaluation, and process improvement. Includes measurement differences; accountability for collection, documentation, and analysis. |  |  |  |  |  |
| **Factor 24**- **Engaging Organizations, External Context (revised):**developing and capitalizing on relationships with healthcare professionals and frontline staff in the various organizations involved in the implementation of a transition care innovation, and promoting external collaborations with other outside care providers (e.g. home care agency), and resources (e.g. community resources or social services for older persons) linked to the implementation. |  |  |  |  |  |
| **Factor 25**- **Engaging Innovation Participants (revised):** individuals (patients/older persons, family, informal caregivers) served by the organization that participate in the transitional care innovation (e.g. ensuring family inclusion in care goals setting). |  |  |  |  |  |
| ***Additional New Factors (suggested by respondents from round 1): New Factor 1 - Power of Decision-makers:** defined as the main trigger to allow the successful implementation of a transitional care innovation. |  |  |  |  |  |
| **New Factor 2 - Sense of Urgency:** the urgent need and attention given to implementing a specific transitional care innovation with respect to other innovation projects being addressed within an organization. |  |  |  |  |  |
| **New Factor 3 - Adoption of Change in Work Processes:** adapting and changing "how things work" across several levels (organizational, group, individual, policy); and in view of the capacity for implementing this change within an organization. |  |  |  |  |  |
| **New Factor 4 - Financing of Transitional Care Innovation** **Implementations:** the existing financing structures that affect the implementation such as fragmented financing & a lack of clear financing structures, or varied reimbursement systems of healthcare services. |  |  |  |  |  |
| **New Factor 5 - Inter-organizational Collaborations:** the presence of long-lasting trust relationships between multiple organizations involved in transitional care; in addition to the level of good collaboration and care coordination among different organizations within different and various sectors of the healthcare system, as well as varied care disciplines/services. |  |  |  |  |  |
| **New Factor 6 - Previous Experiences with Implementation of Innovations:** a prior history and experience of organizations & healthcare professionals in implementing change and new interventions, programs, and innovations. |  |  |  |  |  |
| **New Factor 7 - Co-design of the Transitional Care Innovation:** the degree of involvement of the stakeholders (older people and healthcare providers) in the design of the innovation prior to the implementation stages. |  |  |  |  |  |

Q1) Please provide any further comments you might have on the importance of influence of the listed factors in relation to implementing Transitional Care Innovations:

________________________________________________________________

________________________________________________________________

________________________________________________________________

________________________________________________________________

________________________________________________________________

**Section II - Feasibility (easiness/difficulty):** the ability to address factors in the development of implementation strategies can be intricate and complex due to the overall context, which can be varied for each innovation implementation. Therefore, utilize your experience in the current healthcare field in order to respond to this section.  
  
The factors listed below are those that did not reach consensus in round 1, in addition to 7 new factors suggested by the respondents from round 1.

>Please, for each factor review the group choices & your individual choice from round 1 and re-rate the **easiness/difficulty** to address each in the development of implementation strategies for Transitional Care Innovations. Also, you may decide to keep the original choice that you had from round 1.

>Experts' comments for some factors from round 1 are provided where available & applicable, in order to clarify further their definition and interpretation. For the new factors, please rate accordingly.

>Group choices are provided as % out of 100% = 29 (total number of respondents in round 1).

>Note: for some factors their definitions were revised for this round(based on experts comments, the definition is rephrased and enhanced to maximize clarity), in this case a label (revised) is added only next to those factors whose definitions were enhanced.

**Please, note that the group choices, individual choices percentages, and experts’ comments were omitted from this additional file, for data protection purposes.*

|  | Very difficult | Difficult | Neither difficult nor easy | Easy | Very easy |
| --- | --- | --- | --- | --- | --- |
| **Domain I - Intervention (Transitional Care Innovation) Characteristics**  **Factor 1 - Targeted Groups (revised):** patients/older population who are the intended recipients or beneficiaries of the transitional care innovation (e.g. matching the care needs of older persons with high frailty or dementia). |  |  |  |  |  |
| **Factor 3 - Relative Advantage:** stakeholders’ perception of the advantage (benefits and usefulness) of implementing the transitional care innovation versus an alternative solution. |  |  |  |  |  |
| **Factor 4 - Evidence Strength & Quality**: stakeholders’ perceptions of the quality and validity of evidence (proven effectiveness) supporting the belief that the transitional care intervention will have desired outcomes (e.g. low readmission rates). |  |  |  |  |  |
| **Domain II - Outer Setting**  **Factor 5 - Cosmopolitanism (revised):** the degree to which an organization is networked with other external organizations (e.g. pre-existing partnerships; sharing of healthcare practices with external organizations). |  |  |  |  |  |
| **Domain III - Inner (Organizational) Setting**  **Factor 9 - Relative Priority (revised):**individuals’ (healthcare professionals, staff within implementing team) shared perception of the importance of the implementation of a transitional care innovation within the organization (e.g. existence of multiple quality improvement projects within the organization at the same time). |  |  |  |  |  |
| **Factor 10 - Leadership Engagement (revised):**commitment, involvement, and accountability of leaders & managers with the implementation of a transitional care innovation. In addition, the presence of a skilled, motivated, and continuous leadership throughout the implementation (e.g. minimal turnover of dedicated project managers with high interest in the implementation). |  |  |  |  |  |
| **Factor 12 - Access to Knowledge & Information:**ease of access to digestible information and knowledge (e.g. mentoring, initial training) about the transitional care innovation and how to incorporate it into work tasks. |  |  |  |  |  |
| **Factor 13 - Continuity (revised):**care transitions' information continuity (e.g. exchange of patient medical information and the services & care plans between healthcare providers). In addition, the continuity of steady work relationships between the healthcare providers and patients/caregivers and across all the organizations involved in the transitional care innovation implementation. |  |  |  |  |  |
| **Domain IV - Characteristics of Individuals**  **Factor 15a - Knowledge & Beliefs about the Intervention: healthcare professionals/staff** within implementing team's beliefs, expectations, and familiarity with facts, truths, & principles related to the transitional care innovation. |  |  |  |  |  |
| **Factor 15b - Knowledge & Beliefs about the Intervention: patients/older persons'** attitudes toward and value placed on the transitional care innovation as well as awareness on its care services & goals. |  |  |  |  |  |
| **Factor 16 - Role:**individual’s role (healthcare professionals, staff within implementing team) and responsibility for the transitional care innovation; including the degree of multiple or shared roles. |  |  |  |  |  |
| **Factor 17 - Skills & Competencies:**degree of relevant subject matter expertise, skills, and competencies within the implementing team, unit, and organization. |  |  |  |  |  |
| **Factor 18b - Other Personal Attributes: patients/older persons'**other personal traits such as health literacy, values, and acknowledgement of own care needs. |  |  |  |  |  |
| **Domain V - (Implementation) Process**  **Factor 20 - Transition Roles (Frontline Staff):** administrative staff, providers (within and outside the organization), e.g. frontline staff such as transition nurses or advanced practice nurses with designated transition roles who will carry out the innovation or be affected by it. |  |  |  |  |  |
| **Factor 21 - Reflecting & Evaluating:**quantitative and qualitative feedback about the progress and quality of implementation accompanied with regular personal and team debriefing about progress and experience. |  |  |  |  |  |
| **Factor 22**- **Measurement Capability/Data Availability:**availability of timely data. Capacity for monitoring, evaluation, and process improvement. Includes measurement differences; accountability for collection, documentation, and analysis. |  |  |  |  |  |
| **Factor 23**- **Engaging Key Stakeholders:**individuals from within the organization that are directly impacted by the transitional care innovation, e.g. staff responsible for making referrals to a new program or using a new work process. |  |  |  |  |  |
| **Factor 24**- **Engaging Organizations, External Context (revised):**developing and capitalizing on relationships with healthcare professionals and frontline staff in the various organizations involved in the implementation of a transition care innovation, and promoting external collaborations with other outside care providers (e.g. home care agency), and resources (e.g. community resources or social services for older persons) linked to the implementation. |  |  |  |  |  |
| **Factor 25**- **Engaging Innovation Participants (revised):**individuals (patients/older persons, family, informal caregivers) served by the organization that participate in the transitional care innovation (e.g. ensuring family inclusion in care goals setting). |  |  |  |  |  |
| ***Additional New Factors (suggested by respondents from round 1):**  **New Factor 1 - Power of Decision-makers:** defined as the main trigger to allow the successful implementation of a transitional care innovation. |  |  |  |  |  |
| **New Factor 2 - Sense of Urgency:** the urgent need and attention given to implementing a specific transitional care innovation with respect to other innovation projects being addressed within an organization. |  |  |  |  |  |
| **New Factor 3 - Adoption of Change in Work Processes:** adapting and changing "how things work" across several levels (organizational, group, individual, policy); and in view of the capacity for implementing this change within an organization. |  |  |  |  |  |
| **New Factor 4 - Financing of Transitional Care Innovation Implementations:** the existing financing structures that affect the implementation such as fragmented financing & a lack of clear financing structures, or varied reimbursement systems of healthcare services. |  |  |  |  |  |
| **New Factor 5 - Inter-organizational Collaborations:** the presence of long-lasting trust relationships between multiple organizations involved in transitional care; in addition to the level of good collaboration and care coordination among different organizations within different and various sectors of the healthcare system, as well as varied care disciplines/services. |  |  |  |  |  |
| **New Factor 6 - Previous Experiences with Implementation of Innovations:** a prior history and experience of organizations & healthcare professionals in implementing change and new interventions, programs, and innovations. |  |  |  |  |  |
| **New Factor 7 - Co-design of the Transitional Care Innovation:** the degree of involvement of the stakeholders (older people and healthcare providers) in the design of the innovation prior to the implementation stages. |  |  |  |  |  |

Q2) Please provide any further comments you might have on the feasibility (easiness/difficulty) to address each of the listed factors in relation to implementing Transitional Care Innovations:

________________________________________________________________

________________________________________________________________

________________________________________________________________

________________________________________________________________

________________________________________________________________
