# Supplementary material for: Fostering the implementation of transitional care innovations for older persons: prioritizing the influencing key factors using a modified Delphi technique
Source: BMC Geriatr. 2022 Feb 16;22:131. doi: 10.1186/s12877-021-02672-2 (PMC8848680; doi:10.1186/s12877-021-02672-2)
Supplement: Supplementary file 4 — Additional file 4. List of factors that reached consensus on direction of influence on the implementation of TCIs, from round one. [file 12877_2021_2672_MOESM4_ESM.docx]

**Additional file 4: List of factors that reached consensus on direction of influence on the implementation of TCIs, from round one**.

| **Factor** | **Rating: Hindering/Strongly Hindering**  (Consensus level in %) | **CFIR Domain** |
| --- | --- | --- |
| ***Round one*** |  |  |
| Complexity | 96 | Intervention characteristics |
|  | **Rating: Facilitating/Strongly Facilitating**  (Consensus level in %) |  |
| Leadership engagement | 100 | Inner setting |
| Engaging key stakeholders | 97 | Process |
| Relative advantage | 93 | Intervention characteristics |
| External incentives | 93 | Outer setting |
| Evidence strength and quality | 90 | Intervention characteristics |
| Relative priority | 90 | Inner setting |
| Available resources | 86 | Inner setting |
| Access to knowledge and information | 86 | Inner setting |
| Transition roles - frontline staff | 86 | Process |
| Skills and competencies | 83 | Characteristics of individuals |
| External policy | 83 | Outer setting |
| Networks and communications | 83 | Inner setting |
| Planning | 79 | Process |
| Reflecting and evaluating | 79 | Process |
| Knowledge and beliefs of healthcare professionals about the TCIs | 76 | Characteristics of individuals |
| Measurement capability/data availability | 72 | Process |
| Engaging organizations, external context | 72 | Process |
